# A Metabolic Phenotype Based on Mitochondrial Ribosomal Protein Expression as a Predictor of Lymph Node Metastasis in Papillary Thyroid Carcinoma: Erratum

**DOI:** 10.1097/01.md.0000464214.86167.22

**Published:** 2015-02-27

In the article “A Metabolic Phenotype Based on Mitochondrial Ribosomal Protein Expression as a Predictor of Lymph Node Metastasis in Papillary Thyroid Carcinoma”,^1^ which appeared in Volume 94, Issue 2 of *Medicine*, several abbreviations were left undefined in figure and table legends. The article has since been corrected online.

## References

[1] Lee J Seol M-Y Jeong S A metabolic phenotype based on mitochondrial ribosomal protein expression as a predictor of lymph node metastasis in papillary thyroid carcinoma. *Medicine* 94 2:e380. 25590838 10.1097/MD.0000000000000380 PMC4602546

